# Prediction of protein-protein interactions between viruses and human by an SVM model

**DOI:** 10.1186/1471-2105-13-S7-S5

**Published:** 2012-05-08

**Authors:** Guangyu Cui, Chao Fang, Kyungsook Han

**Affiliations:** 1 School of Computer Science and Engineering, Inha University, Incheon, South Korea

## Abstract

**Background:**

Several computational methods have been developed to predict protein-protein interactions from amino acid sequences, but most of those methods are intended for the interactions within a species rather than for interactions across different species. Methods for predicting interactions between homogeneous proteins are not appropriate for finding those between heterogeneous proteins since they do not distinguish the interactions between proteins of the same species from those of different species.

**Results:**

We developed a new method for representing a protein sequence of variable length in a frequency vector of fixed length, which encodes the relative frequency of three consecutive amino acids of a sequence. We built a support vector machine (SVM) model to predict human proteins that interact with virus proteins. In two types of viruses, human papillomaviruses (HPV) and hepatitis C virus (HCV), our SVM model achieved an average accuracy above 80%, which is higher than that of another SVM model with a different representation scheme. Using the SVM model and Gene Ontology (GO) annotations of proteins, we predicted new interactions between virus proteins and human proteins.

**Conclusions:**

Encoding the relative frequency of amino acid triplets of a protein sequence is a simple yet powerful representation method for predicting protein-protein interactions across different species. The representation method has several advantages: (1) it enables a prediction model to achieve a better performance than other representations, (2) it generates feature vectors of fixed length regardless of the sequence length, and (3) the same representation is applicable to different types of proteins.

## Background

A variety of viruses cause diseases in humans, and viral infections affect millions of people each year. The treatment and prevention of viral infections and their associated diseases are the main public health challenges. Common examples of viruses associated with human diseases include HIV-1, influenza virus, human papillomavirus (HPV), herpes virus, and hepatitis A, B, C, D and E viruses. Different viral species have different infection mechanisms, and identifying host cell proteins that are attacked by a virus will certainly help better understand the infection mechanism and to design new antiviral strategies. Recently, proteome-wide studies of viral interactions with human proteins were performed, but comprehensive analysis of the interactions between virus proteins and human proteins involved in viral infection has not yet been investigated.

So far, most computational studies of protein-protein interactions (PPIs) have focused on the interactions between proteins of the same species. For example, Bock and Gough [1] created protein structural and physiochemical descriptors based on the sequence data, and trained a support vector machine classifier to identify PPIs from the descriptors. There are many other studies that used a support vector machine to predict PPIs in several organisms [2-6].Wu *et al*. [7] used the Gene Ontology (GO) and other annotations to predict PPIs in yeast. Nanni [8] predicted PPIs in the human gastric bacterium *Helicobacter pylori *by combining a linear discriminant classifier and cloud points. You *et al*. [9] used a manifold embedding method to assess and predict PPIs. All these methods are intended for the protein-protein interactions within a species rather than for those across different species. Methods typically used to predict interactions between homogeneous proteins are not appropriate for predicting those between heterogeneous proteins, since such methods do not distinguish interactions between proteins of the same species from those of different species.

In this paper, we propose a representation method and a support vector machine (SVM) model to predict the interactions between two types of viruses (hepatitis C virus and human papillomaviruses) and human proteins. Although substantial progress has been made in clinical and basic research on the hepatitis C virus (HCV) and human papillomaviruses (HPV), interactions between these viruses and human proteins are not yet fully understood. Identifying more interactions between them should help elucidate the interaction mechanism of HCV and HPV with host cells, and can be helpful in designing molecules that target the new interacting proteins.

## Methods

### Representation of protein sequences

One of the challenges in predicting protein-protein interactions from sequences is to find a way of fully encoding the important information content of protein sequences. In addition, the amino acid sequences of different lengths should be transformed into a feature vector of the same length. We represent a protein sequence using three consecutive amino acids called *amino acid triplet*. For example, in the amino acid sequence TVAVTVA, there are four overlapping amino acid triplets, TVA, VAV, AVT and VTV. To reduce the dimension of the vector space of feature vectors, we represent an amino acid sequence using the class of amino acids. Based on the biochemical similarity of amino acids, twenty amino acids were classified into six categories: {IVLM}, {FYW}, {HKR}, {DE}, {QNTP}, and {ACGS} [10,11]. Using this classification of amino acids, there are 6×6×6 = 216 possible amino acid triplets.

We use a binary space (V, F) to represent a protein sequence, in which V is a vector space of feature vectors with a fixed number of features and F is a vector space of frequency vectors. A protein sequence of variable length is first mapped to a feature vector of fixed length. A feature vector *v *is then mapped to a relative frequency vector *d_i _*(*i*=1, 2, ..., 216), which is defined by equation 1.

(1) $$d_{i}=\left\{e^{\frac{fi-\min\{f_{1},f_{2},...,f_{216}\}}{\max\{f_{1},f_{2},...,f_{216}\}-\min\{f_{1},f_{2},...,f_{216}\}}}\right\}-1$$

where *f_i _*is the frequency of the *i-*th triplet type in the sequence. The value of *d_i _*ranges from 0 to 1.714.

There are two differences between our representation and that of Shen *et al*. [5], namely in the classification of amino acids and in the definition of the relative frequency of an amino acid triplet. First, Shen *et al*. [5] clustered the 20 amino acids into seven classes, {AVG}, {ILFP}, {YMTS}, {HNQW}, {RK}, {DE}, {C}, based on the diploes and volumes of the side chains of amino acids, and there are 7×7×7 = 343 possible amino acid triplets. Second, the relative frequency of a triplet in their representation is defined by equation 2.

(2) $$d_{i}=\frac{f_{i}-\text{min}\left\{f_{1},f_{2},\ldots ,f_{343}\right\}}{\text{max}\left\{f_{1},f_{2},\ldots ,f_{343}\right\}-\text{min}\left\{f_{1},f_{2},\ldots ,f_{343}\right\}}$$

While the relative frequency in the representation of Shen *et al*. [5] has a value in the range 0[1], it ranges from 0 to 1.714 in our representation. Thus, the relative frequency value in a wider range makes it easier to discriminate protein sequences as we will show later in the results section.

In addition to the relative frequencies of amino acid triplets, a feature element representing the types of virus proteins (11 types of HCV proteins and 9 types of HPV proteins) were included in a feature vector. Each feature vector was labelled +1 for interaction and -1 for non-interaction. By encoding the type of a virus protein, the SVM model can find a human protein interacting with the virus protein.

### Support vector machine

A support vector machine (SVM) has been applied to several biological problems such as prediction of protein-protein interactions [1-6], homology detection [12], and analysis of gene expression data [13]. Data examples labelled positive or negative are projected into a high-dimensional feature space using a kernel, and the hyper-plane in the feature space is optimized to maximize the margin between positive and negative data examples. We implemented an SVM model using LIBSVM http://www.csie.ntu.edu.tw/~cjlin/libsvm/ with the radial basis function (RBF) as a kernel function, which is defined by

(3) $$K\left(u,v\right)=\text{exp}\left(-\gamma ||u-v|{|}^{2}\right)$$

where *u *and *v *are two data vectors and γ is a training parameter. A smaller γ value makes the decision boundary smoother. The regularization factor C controls trade-off between a low training error and a large margin [14]. In this study, we set C = 20 and γ=0.1.

We tried several other kernel functions with our data. The linear and polynomial kernel functions resulted in high sensitivity (almost 100%), but low specificity (about 50%). The sigmoid kernel function showed poor performance both in sensitivity and specificity (about 50%). The radial basis function was the only one that showed reasonably good sensitivity and specificity, and chosen as the kernel function of the SVM model.

### Data set of viral interaction with human proteins

Hepatitis C virus (HCV) is a small enveloped virus with a single-stranded RNA genome encoding a single open reading frame [15]. The polyprotein of approximately 3,100 amino acids is cleaved into the structural proteins (core, E1 and E2), hydrophobic peptide p7, and non- structural proteins such as NS2, NS3, NS4A, NS4B, NS5A and NS5B [16]. Although many experimental studies have been performed so far, the underlying mechanisms controlling the entry of HCV into host cells and interactions with the host cells are not fully known, and an efficient treatment for HCV infection has not yet been developed.

We obtained the interaction data between HCV proteins and human proteins from the infection mapping project (I-MAP) [17]. I-MAP provides a comprehensive view of viral infections at the protein level by mapping the interactions of a large amount of viral proteins with host proteins. There are 481 interactions between 11 HCV proteins and 421 human proteins. By searching Gene IDs of the 421 human proteins in HPRD http://www.hprd.org, we identified a total of 695 interactions between HCV proteins and human proteins. The 695 protein-protein interactions formed a positive data set for an SVM model. Both positive and negative data are required to train an SVM model. Unlike positive data, negative samples are not readily available from protein-protein interaction data. We randomly selected 695 human proteins from HPRD, which were not included in the positive data set, and generate a negative data set with them. Our method of generating a negative data set is similar to that of Gomez *et al*. [10], which assumes a negative protein interaction if there is no explicit evidence of an interaction. Since an unbalance between positive and negative data sets introduces a prediction bias, we generated a negative data set with the same number of proteins as the positive data set.

For evaluating an SVM model, we divided both the positive and negative data sets into training and test sets. We randomly selected 500 positive data and 500 negative data for a training set. The remaining 195 positive data and 195 negative data were used to construct a test set. To keep the same proportion of human proteins interacting with each virus protein in both training and test sets, we selected training data by

(4) $$N_{i}=N\left(Training\right)\cdot \frac{N\left(T_{i}\right)}{N\left(Total\right)}$$

where *T_i _*is the *i-*th virus protein (*i*=1, 2, ..., 11 in HCV), N(*T_i_*) is the number of human proteins interacting with the *i-*th HCV protein, N(Training) is the total number of positive training data, and N(Total) is the total number of HCV-human protein interactions.

Table 1 shows the numbers of human proteins known to interact with each HCV protein, and those selected for a training set. For example, 298 human proteins are known to interact with the HCV NS3 protein. 214 out of the 298 human proteins were randomly selected as positive data of a training set since N(NS3) = 500 × 298 / 695 = 214. We selected the same number of human proteins from a negative dataset as negative interaction partners of the HCV NS3 protein.

**Table 1:** The number of human proteins interacting with HCV proteins

HCV protein	Number of H_HCV _	Number of H_HCV _in a training set
core	118	85
E1	16	12
E2	29	21
F	10	7
NS2	11	8
NS3	298	214
NS4A	7	5
NS4B	1	1
NS5A	152	109
NS5B	36	26
p7	17	12

Total	695	500

H_HCV _represents the human proteins that are known to interact with HCV proteins. For each HCV protein, the number of H_HCV _in a training set was computed by $N_{i}=N\left(Training\right)\cdot \frac{N\left(T_{i}\right)}{N\left(Total\right)}$, where *T_i _*is the *i-*th HCV protein.

Human papillomavirus (HPV) is a member of the papillomavirus family of viruses that is capable of infecting humans. HPV types 16 and 18 cause 70% of cervical cancer [18,19]. So far, a small number of host proteins have been known to interact with HPV proteins, so a systematic prediction of large-scale interactions between HPV proteins and human proteins would help find new human proteins targeted by HPV. We extracted the interactions of HPV-16 and HPV-18 proteins with human proteins from the NCBI BioSystems Database (http://www.ncbi.nlm.nih.gov/biosystems/). After removing redundancy, we identified a total of 252 interactions of HPV proteins with human proteins, and obtained Gene IDs from HPRD http://www.hprd.org. A training set and test set for HPV interactions were constructed in the same way as for the HCV interactions. Table 2 shows the numbers of human proteins known to interact with each HPV protein and those used to train an SVM model for HPV interactions.

**Table 2:** The number of human proteins interacting with HPV proteins

HPV protein	Number of H_HPV_	Number of H_HPV _in a training set
E1	9	7
E2	36	29
E4	2	2
E5	13	10
E6	78	62
E7	76	60
E8	7	6
L1	20	16
L2	11	8

Total	252	200

H_HPV_ represents the human proteins that are known to interact with HPV proteins. For each HPV protein, the number of H_HPV_ in a training set was computed by $N_{i}=N\left(Training\right)\cdot \frac{N\left(T_{i}\right)}{N\left(Total\right)}$ , where **T_i _**is the **i-**th HPV protein.

## Results and discussion

### Performance evaluation

We evaluated the performance of the SVM model in terms of sensitivity, specificity and accuracy.

(5) $$Sensitivity=\frac{TP}{TP+FN}$$

(6) $$Specificity=\frac{TN}{TN+FP}$$

(7) $$Accuracy=\frac{TP+TN}{TP+FP+TN+FN}$$

True positives (TP) are actual interacting proteins that are predicted correctly. True negatives (TN) are non-interacting proteins that are predicted correctly. False positives (FP) are non-interacting proteins that are predicted as interacting proteins. False negatives (FN) are interacting proteins that are missed.

We did not perform cross validation to evaluate the SVM model. Instead, we prepared independent test sets that were not used in training the SVM model. In general, cross-validation shows a much higher performance than testing a prediction model on a new data set that was not used in training. As shown later in this paper, some virus proteins have a very small number of human protein interaction partners to perform cross validation. For example, the HCV NS4A and HCV NS4B proteins have only 7 and 1 interaction partners, respectively. The HPV E4 protein has only 2 interaction partners, and the HPV E8 protein has only 7 interaction partners. Thus, we tested the SVM model on new, independent data sets that were not used in training the model.

### Interaction of HCV proteins

Due to the randomness in drawing negative data from HPRD and positive data from the data set of HCV-human protein interactions for the training set, we prepared three independent test sets and evaluated the SVM model with the sets (Additional file 1). For comparison, we also tested the method of Shen *et al*. [5] on these sets. Table 3 compares our method with Shen's method in terms of sensitivity, specificity and accuracy with the HCV data.

**Table 3:** Comparison of two representation methods for predicting human proteins interacting with HCV proteins

Test set	Our representation			Shen's representation		
	
	SN (%)	SP (%)	AC (%)	SN (%)	SP (%)	AC (%)
1	75.9	83.6	79.7	73.8	82.1	77.9
**2**	**80.5**	**89.7**	**85.1**	**73.8**	**82.1**	**77.9**
3	76.9	83.1	80	74.4	76.9	75.6

Average	77.8	85.4	81.6	74.0	80.4	77.1

Comparison of our representation method with the method by Shen *et al*. [5] in terms of sensitivity (SN), specificity (SP) and accuracy (AC) with the HCV data. The two representation methods are different in their classification of 20 amino acids and definition of the relative frequency of an amino acid triplet (see Methods section for details).

As shown in Table 3, our SVM model, on average, achieved a sensitivity of 77.8%, a specificity of 85.4% and an accuracy of 81.6%. It outperformed the method of Shen *et al*. [5], which on average, achieved a sensitivity of 74.0%, a specificity of 80.4% and an accuracy of 77.1%. In particular, our method showed the best performance in the second test set.

To find new human proteins that potentially interact with HCV proteins (H_HCV_), we ran BLAST http://www.ncbi.nlm.nih.gov/BLAST/ with the known H_HCV _as query sequences against the human proteins in GenBank http://www.ncbi.nlm.nih.gov/genbank/. The E-value was set to 10^-20 ^when running BLAST. After removing redundant sequences with the 695 known H_HCV _proteins, we obtained a total of 4,209 human proteins as the initial candidates of H_HCV _(Table 4).

**Table 4:** New human proteins found by our method as potential interaction partners with HCV proteins

HCV protein	Known H_HCV_	Initial candidates of H_HCV _by BLAST search	Predicted candidates of H_HCV _by SVM	Refined candidates of H_HCV _with GO
core	118	732	225	71
E1	16	150	28	9
E2	29	182	33	10
F	10	206	57	12
NS2	11	176	42	8
NS3	298	1,599	495	195
NS4A	7	114	33	10
NS4B	1	1	1	1
NS5A	152	499	123	72
NS5B	36	384	92	51
p7	17	166	51	17

Total	695	4,209	1,180	456

The 'Initial candidates of H_HCV _by BLAST search' indicate the initial candidates of human proteins interacting with HCV proteins (called H_HCV _in this paper) found by BLAST search from GenBank with the known H_HCV _as query sequences. The 'Predicted candidates of H_HCV _by SVM' were determined by the SVM model from the initial candidates of H_HCV_. The 'Refined candidates of H_HCV _with GO' were obtained from the predicted candidates by selecting H_HCV _that have the same GO cellular component terms as the known H_HCV._

**Figure 1:**
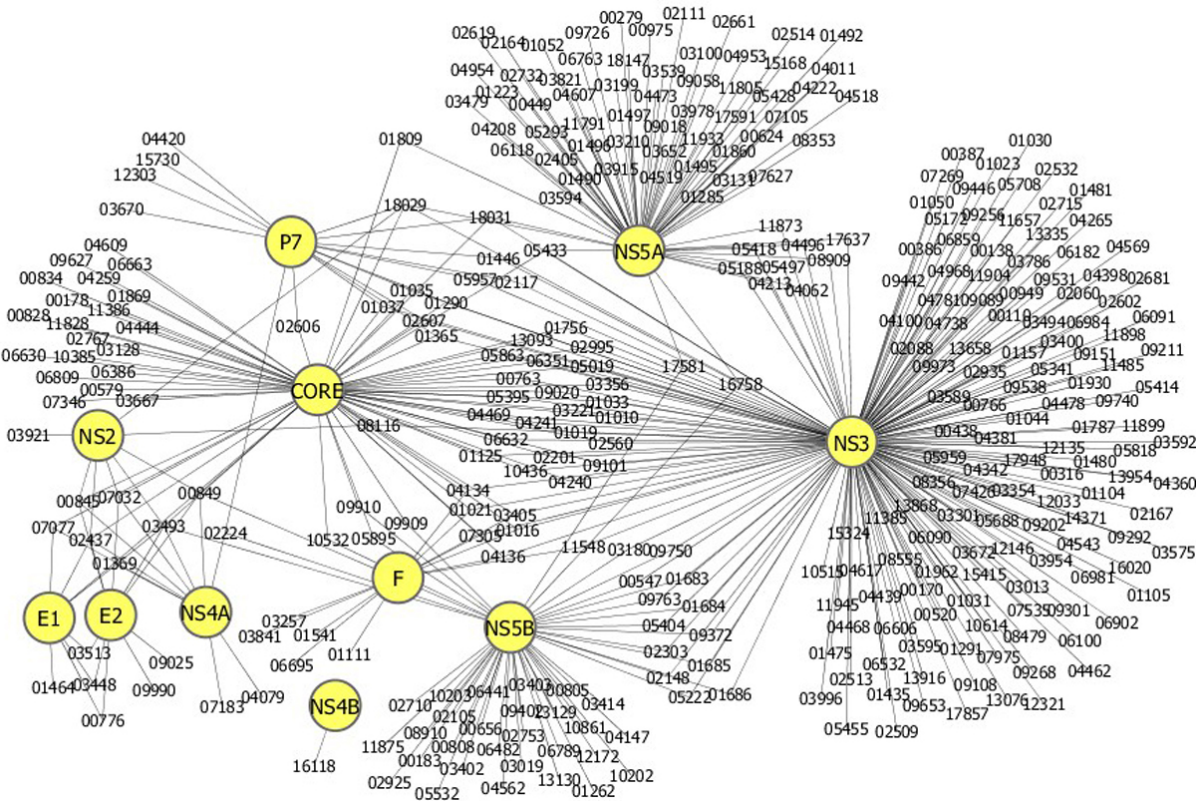
**A network of the human-HCV protein interactions predicted by our method**. The network visualized by Cytoscape [21] includes 11 HCV proteins (core, E1, E2, F, NS2, NS3, NS4A, NS4B, NS4A, NS5B, and p7) and 456 human proteins. The HCV proteins are represented by yellow nodes, and human proteins are shown by nodes with HPRD IDs.

In the 4,209 human proteins, the SVM model predicted 1,180 proteins as potential candidates of H_HCV_. The 1,180 candidates of H_HCV _were refined further by selecting human proteins that have the same gene ontology (GO) cellular component terms [20] as the known H_HCV _for each HCV protein. After this refinement, we obtained a total of 456 candidates of H_HCV_. For instance, the HCV E2 protein has 29 known H_HCV _proteins, and the 29 H_HCV _proteins have a total of 15 GO cellular component terms. The SVM model predicted 33 H_HCV _proteins as interacting partners of the HCV E2 protein, and 10 out of the 33 candidates were left as reliable candidates of H_HCV_, since they have the same GO cellular component terms as the known H_HCV _proteins. Figure 1 shows an interaction network of the 456 H_HCV _proteins predicted by our method.

### Interaction of HPV proteins

To evaluate the performance of the model with the HPV datasets, we prepared three training sets and three test sets (Additional file 2). We tested both our method and Shen's method [5] on the test sets. As shown in Table 5, our method achieved on average, a sensitivity of 78.8%, a specificity of 87.8% and an accuracy of 83.3%. Shen's method showed, on average, a sensitivity of 72.4%, a specificity of 83.9% and an accuracy of 78.2%. In both HCV and HPV data sets, our method was better than Shen's method. The major difference between our method and Shen's method is in the representation of protein sequences. Our classification of 20 amino and definition of the relative frequency of an amino acid triplet are different from those of Shen's method (see the Methods section for details).

**Table 5:** Comparison of two methods for predicting human proteins interacting with HPV proteins

Test set	Our representation			Shen's representation		
	
	SN (%)	SP (%)	AC (%)	SN (%)	SP (%)	AC (%)
**1**	**86.5**	**88.5**	**87.5**	**69.2**	**84.6**	**76.9**
2	73.1	88.5	80.8	69.2	84.6	76.9
3	76.9	86.5	81.7	78.8	82.7	80.8

Average	78.8	87.8	83.3	72.4	83.9	78.2

Comparison of our representation method with the method by Shen *et al*. [5] in terms of sensitivity (SN), specificity (SP) and accuracy (AC) with the HPV data.

**Figure 2:**
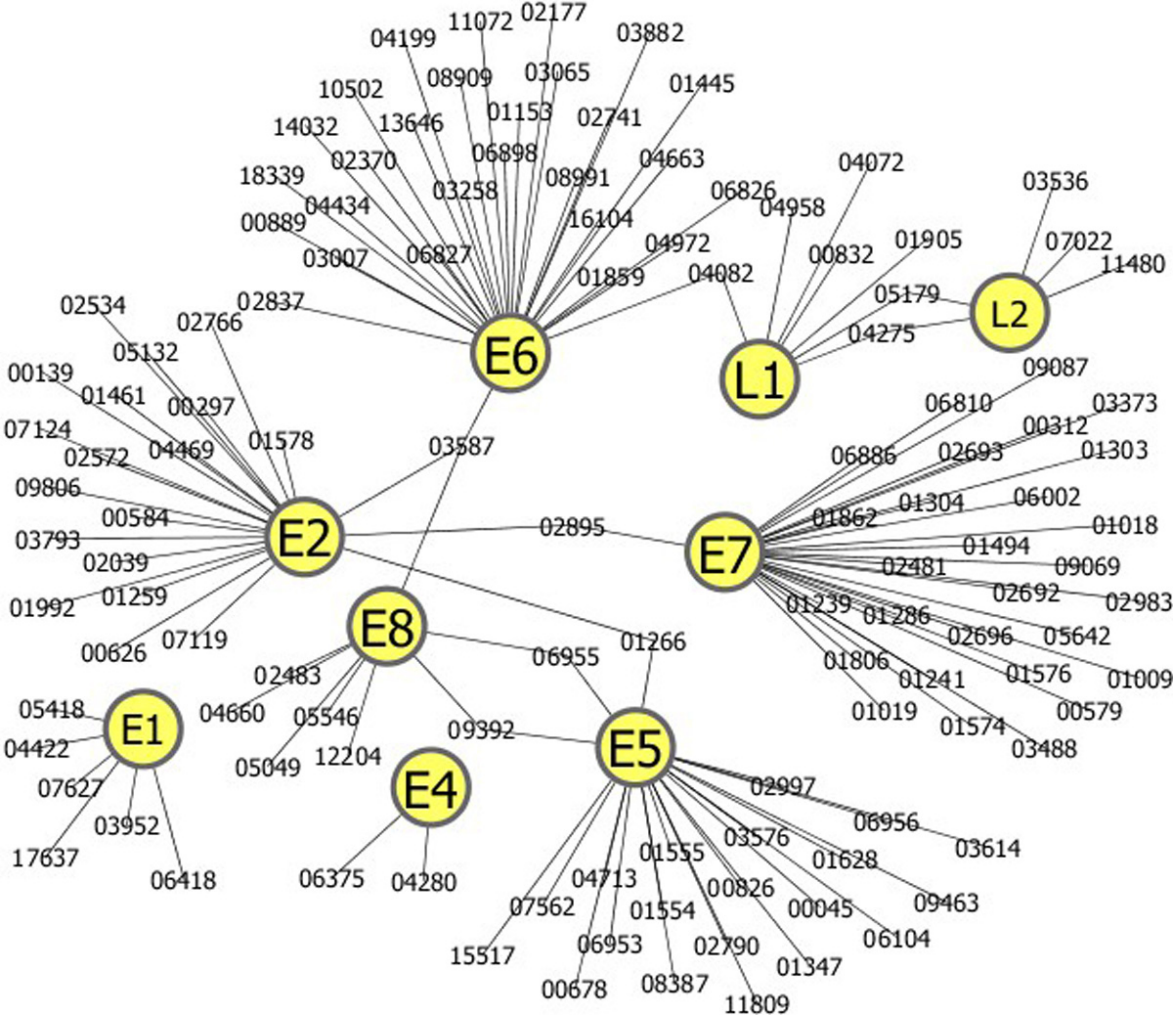
**A network of the human-HPV protein interactions predicted by our method**. The network includes 9 HPV proteins (E1, E2, E4, E5, E6, E7, E8, L1, L2) and 130 human proteins. The HPV proteins and human proteins are represented by yellow nodes and HPRD IDs, respectively.

To find new human proteins that potentially interact with HPV proteins (H_HPV_), we ran BLASTP http://www.ncbi.nlm.nih.gov/BLAST/ with the E-value ≤ 10^-20 ^against the human proteins in GenBank http://www.ncbi.nlm.nih.gov/genbank/. After removing redundant sequences with the 252 known H_HPV _proteins, we obtained a total of 560 human proteins as the initial candidates of H_HPV _(Table 6). In the 560 H_HPV _proteins, the SVM model predicted 156 proteins as potential candidates of H_HPV_. In the 156 H_HCV _proteins, we selected the human proteins that have the same cellular component GO IDs as the known H_HPV _for each HPV protein. As a result, we found a total of 130 human proteins as potential H_HPV_. Figure 2 shows an interaction network of 130 H_HPV _proteins that were predicted by our method.

**Table 6:** New human proteins found by our method as potential interaction partners with HPV proteins

HPV protein	Known H_HPV_	Initial candidates of H_HPV _by BLAST search	Predicted candidates of H_HPV _by SVM	Refined candidates of H_HPV _with GO
E1	9	90	7	6
E2	36	157	28	21
E4	2	11	2	2
E5	13	34	25	23
E6	78	103	38	29
E7	76	100	35	29
E8	7	19	8	8
L1	20	39	8	7
L2	11	7	5	5

Total	252	560	156	130

The 'Initial candidates of H_HPV_ by BLAST search' indicate the initial candidates of human proteins interacting with HPV proteins (H_HPV_) found by BLAST search from GenBank with the known H_HCV_ as query sequences. The 'Predicted candidates of H_HPV_ by SVM' were determined by the SVM model from the initial candidates of H_HPV_. The 'Refined candidates of H_HPV_ with GO' were obtained from the predicted candidates by selecting H_HCV_ that has the same GO cellular component terms as the known H_HPV._

### Comparative analysis of two viral interaction networks

**Figure 3:**
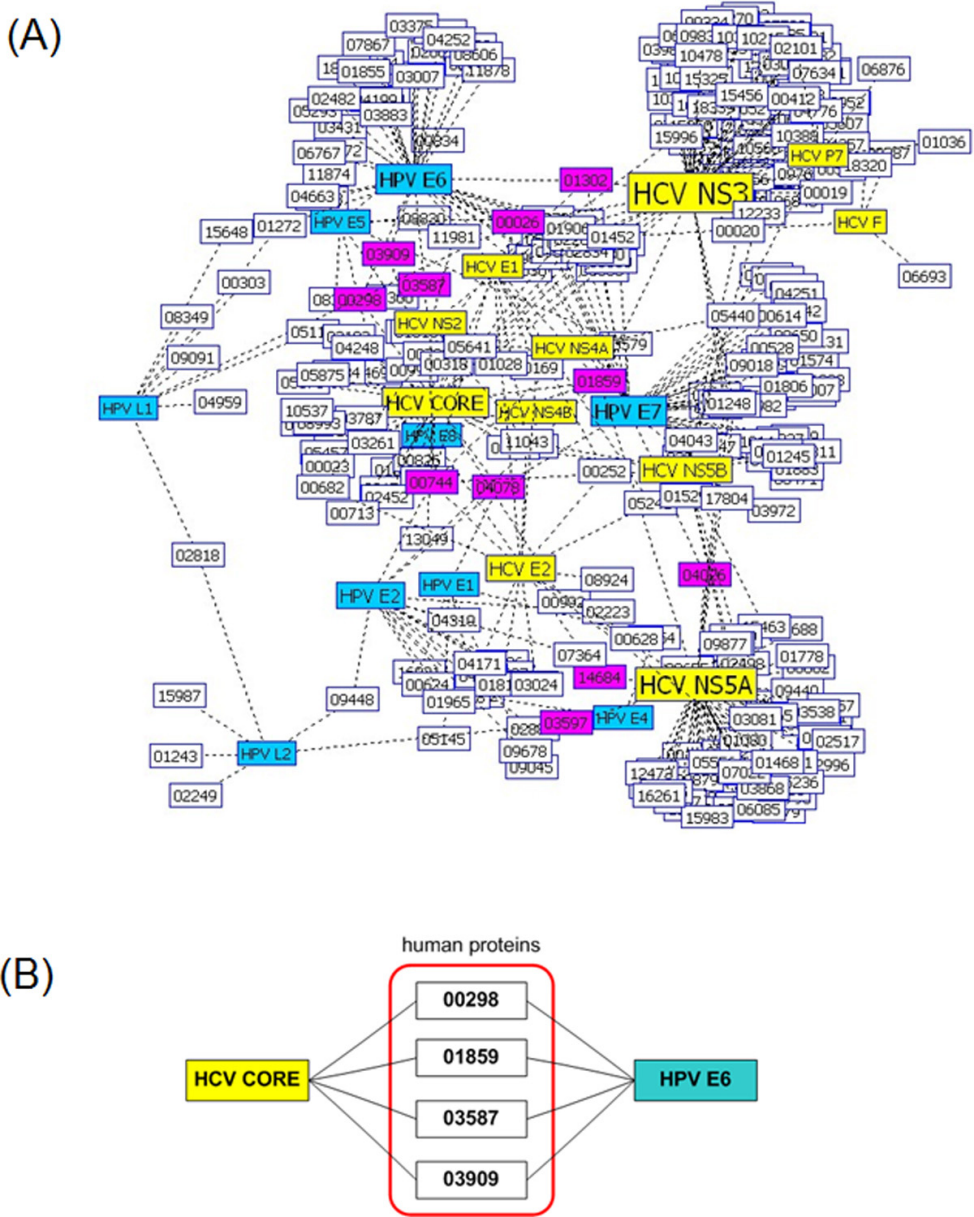
**Comparison of the HCV interaction network *with *the HPV interaction network for the known interactions**. (A) The HCV interaction network is *overlaid by *the HPV interaction network by WebInterViewer [22]. Both networks show the known interactions of HCV and HPV with human proteins. HCV and HPV have 11 human proteins as their common interaction partners: STAT3 (HPRD 00026), CDKN1A (HPRD 00298), NR4A1 (HPRD 00744), JUN (HPRD 01302), TP53 (HPRD 01859), TP73 (HPRD 03587), IPO5 (HPRD 03597), FADD (HPRD 03909), FHL2 (HPRD 04026), EP300 (HPRD 04078), and AHNAK (HPRD 14684). Pink node: human protein interacting with both HCV and HPV proteins; yellow node: HCV protein, cyan node: HPV protein; white node: human protein interacting with either HCV protein or HPV protein but not both. (B) The *CDKN1A (*HPRD 00298*)*, TP53 (HPRD 01859), TP73 (HPRD 03587), and FADD (HPRD 03909) proteins in the red box are the common interaction partners of the HCV core protein and HPV E6 protein. They are known to be engaged in the p53 signaling pathway for apoptosis http://www.sabiosciences.com/rt_pcr_product/HTML/PAHS-027A.html.

In viral infections, different viruses target different human proteins, so they usually have interaction partners. We compared the interaction network of HCV with that of HPV to find common human protein targets. Figure 3A shows the HCV interaction network overlaid by the HPV interaction network, both for the known interaction data. HCV and HPV have 11 human proteins as their common interaction partners: STAT3 (HPRD 00026), CDKN1A (HPRD 00298), NR4A1 (HPRD 00744), JUN (HPRD 01302), TP53 (HPRD 01859), TP73 (HPRD 03587), IPO5 (HPRD 03597), FADD (HPRD 03909), FHL2 (HPRD 04026), EP300 (HPRD 04078), and AHNAK (HPRD 14684). In particular, four human proteins, CDKN1A (HPRD 00298), TP53 (HPRD 01859), TP73 (HPRD 03587), and FADD (HPRD 03909) proteins, shown in Figure 3B, are the common interaction partners of the HCV core protein and HPV E6 protein. They are known to be engaged in the p53 signalling pathway for apoptosis http://www.sabiosciences.com/rt_pcr_product/HTML/PAHS-027A.html.

**Figure 4:**
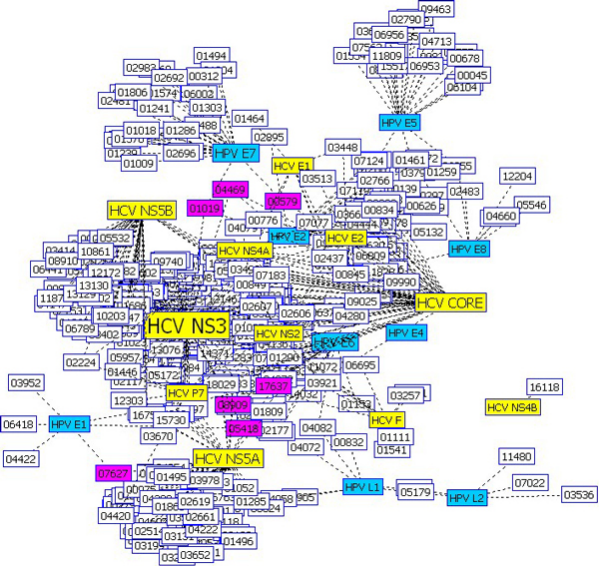
**Comparison of the HCV interaction network *with *the HPV interaction network for the predicted interactions**. The HCV interaction network is *overlaid by *the HPV interaction network by WebInterViewer [22]. Both networks show the predicted interactions of HCV and HPV with human proteins. HCV and HPV have 7 human proteins as their common interaction partners (pink nodes in the network): SLC1A1 (HPRD 00597), KRT17 (HPRD 01019), TP63 (HPRD 04469), GSK3B (HPRD 05418), CDK19 (HPRD 07627), CDK11B (HPRD 08909), and NLK (HPRD 17637). Pink node: human protein interacting with both HCV and HPV proteins; yellow node: HCV protein, cyan node: HPV protein; white node: human protein interacting with either HCV protein or HPV protein but not both.

In a similar way, we compared the predicted interaction network of HCV and that of HPV (Figure 4). HCV and HPV have 7 human proteins as their common interaction partners (pink nodes in the network): SLC1A1 (HPRD 00597), KRT17 (HPRD 01019), TP63 (HPRD 04469), GSK3B (HPRD 05418), CDK19 (HPRD 07627), CDK11B (HPRD 08909), and NLK (HPRD 17637).

## Conclusions

Most methods for predicting protein-protein interactions focus on the interactions within a species rather than for the interactions across different species, such as interactions between virus and host cell proteins. In this paper we presented a support vector machine (SVM) model and its representation method for predicting the interactions between viruses and human proteins.

We represented a protein sequence using three consecutive amino acids called amino acid triplet. We mapped a protein sequence of variable length to a feature vector of fixed length, and then mapped the feature vector to a frequency vector that represents the relative frequency of each triplet within the protein sequence. The SVM model showed an average accuracy of 81.6% in predicting human proteins that interact with HCV proteins, and a similar accuracy (83.3%) in predicting human proteins that interact with HPV proteins. The performance of our SVM model was better than that of the other method [5] in both data sets of HCV and HPV. Using the SVM model and Gene Ontology (GO) annotations of proteins, we also predicted new human proteins that potentially interact with HCV or HPV proteins. From the comparative analysis of two viral interaction networks, we found common human proteins that are targeted by both viruses.

Our experiment on 2 different types of viruses showed that encoding the relative frequency of amino acid triplets of a protein sequence is a simple yet powerful representation method for protein sequences when finding protein-protein interactions across different species. The representation method has several advantages. First, it enables a prediction model to achieve better performance than that of other representations. Second, it generates feature vectors of fixed length regardless of the sequence length. Third, the same representation is applicable to different types of proteins.

Elucidating virus-host interactions is important for understanding viral infections and for identifying new targets for rational drug discovery. In the future, we plan to construct virus-host protein interaction networks to achieve more viral species and perform further comparative analysis of the interaction networks to discover interaction patterns central or specific to them.

## Competing interests

The authors declare that they have no competing interests.

## Authors' contributions

Chao Fang did the early work with HCV data, and Guangyu Cui finished the work with the HPV data and prepared the first draft of the manuscript. Kyungsook Han supervised the work and rewrote the manuscript.

## Supplementary Material

Additional file 1 **Three data sets used for predicting human proteins interacting with HCV proteins**. Three training and test sets of human proteins (HPRD IDs) that were used for the results of Table 3. Click here for file

Additional file 2 **Three data sets used for predicting human proteins interacting with HPV proteins**. Three training and test sets of human proteins (HPRD IDs) that were used for the results of Table 5. Click here for file
